# Endoscopic synchronous injection and submucosal dissection for large granular mixed nodular laterally spreading tumor in the rectum

**DOI:** 10.1055/a-2752-9827

**Published:** 2026-01-08

**Authors:** Xiao-Bo Liu, Hai-Tao Shen, Ruo-Jin Yu, Tie-Yan Wang, Yuan-Jun Gao

**Affiliations:** 1 107632 Department of Gastroenterology, Taihe Hospital, Hubei University of Medicine, Shiyan, China; 2 Department of Respiratory and Critical Care Medicine, Yangquan First People’s Hospital, Yangquan, China; 3 74765 First Clinical College, Hubei University of Medicine, Shiyan, China; 4 107632 Department of Pathology, Taihe Hospital, Hubei University of Medicine, Shiyan, China

**Fig. 1:**
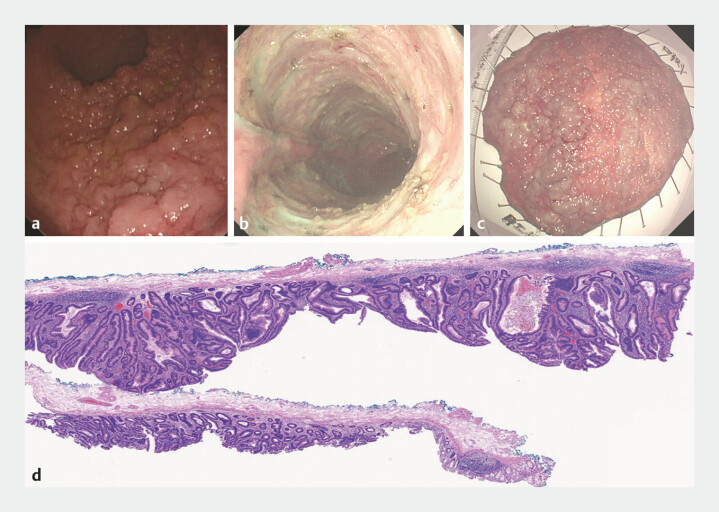
Patient underwent endoscopic mucosal dissection (ESD) and postoperative pathological result. **a** Colonoscopy revealed a nearly 80% circumferential flat elevated lesion located approximately 10 cm proximal to the anal verge and extending to the dentate line, consistent with LST-G-M (laterally spreading tumor, granular mixed subtype). **b** Post-ESISD surgical wound bed. **c** Gross specimen dimensions: 11.5 cm × 11.5 cm. **d** Histopathological examination of the ESD specimen confirmed tubulovillous adenoma with predominantly low-grade dysplasia and focal areas of high-grade dysplasia (hematoxylin and eosin stain, original magnification ×10).

An 81-year-old male patient was admitted for endoscopic submucosal dissection (ESD) of a granular mixed nodular laterally spreading tumor (LST-G-M; Fig. 1 **a** ). The lesion presented as a flat, elevated mass occupying nearly 80% of the rectal circumference, located approximately 10 cm from the anal verge and extending to the dentate line. Given the large lesion size and the rectum’s rich vascular supply, we innovatively employed endoscopic synchronous injection and submucosal dissection (ESISD) to minimize bleeding and improve dissection efficiency. The procedure was completed in 116 minutes, with an estimated blood loss of 10 mL, yielding an en bloc resection specimen measuring 11.5 cm × 11.5 cm. Postoperative pathology revealed tubulovillous adenoma with low-grade dysplasia in most areas and focal high-grade dysplasia ( Fig. 1 **b–d** ). A comparable case using conventional ESD (CESD) served as the control ( Video 1 ).

Endoscopic submucosal injection synchronous dissection without prior needle-assisted injection for the rectal laterally spreading tumor (LST).Video 1

ESD provides a minimally invasive approach for the curative treatment of benign, precancerous, and early neoplastic lesions in the gastrointestinal tract 1 . In CESD, submucosal injection creates a “fluid cushion” that separates the mucosal layer from the muscularis propria, thereby reducing perforation risks 2 . However, rapid absorption of normal saline often requires repeated injections, which can prolong the procedure time. Despite the growing use of novel submucosal injection agents, their cost-effectiveness, accessibility, and the advancement of innovative injection techniques remain critical considerations.

**Fig. 2:**
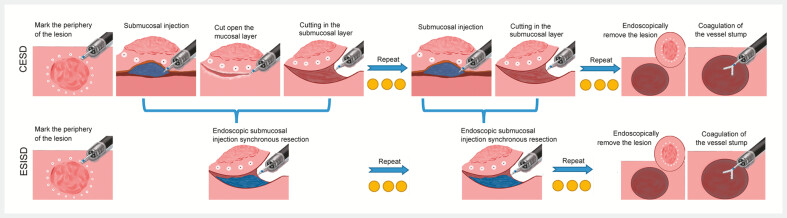
Comparative schematic illustrations of two distinct endoscopic resection techniques: endoscopic submucosal dissection (ESD) and endoscopic submucosal injection synchronous dissection (ESISD).

By contrast, ESISD eliminates the need for injection needles or specialized submucosal agents by integrating injection and dissection into a single procedure ( Fig. 2 ). This approach reduces procedural costs, shortens the operation time, and enhances hemostasis during dissection. Additionally, it minimizes electrosurgical carbonization of the knife, preserving its cutting efficacy. The simplicity and broad applicability of ESISD can enhance traditional ESD workflows, warranting further validation across diverse patient populations.

Endoscopy_UCTN_Code_TTT_1AQ_2AD_3AD
